# Human zoonotic tuberculosis and livestock exposure in low‐ and middle‐income countries: A systematic review identifying challenges in laboratory diagnosis

**DOI:** 10.1111/zph.12684

**Published:** 2020-01-09

**Authors:** Sarah Anne Luciano, Amira Roess

**Affiliations:** ^1^ Department of Environmental and Occupational Health The George Washington University Washington District of Columbia; ^2^ Department of Global and Community Health George Mason University Fairfax Virginia; ^3^ Department of Global Health The George Washington University Washington District of Columbia

**Keywords:** humans, livestock, *Mycobacterium bovis*, ruminants, tuberculosis, zoonosis

## Abstract

**Background:**

Zoonotic tuberculosis (zTB) accounts for 1.4% of the global tuberculosis burden, with the largest disease burden in low‐ and middle‐income countries (LMICs). These populations have increased exposure to zTB due to livestock rearing practices and raw dairy consumption. This qualitative systematic literature review evaluates the quality of the literature that examines the association between human zTB in LMICs and frequent exposure to livestock and livestock products and summarizes current gaps in laboratory detection methods.

**Methods:**

The Navigation Guide, a systematic review framework utilized to assess environmental health exposures, was used to conduct this literature review. Peer‐reviewed research articles were selected and evaluated for risk of bias and quality of evidence. Only studies conducted in LMICs that mentioned livestock or livestock product exposure and had a confirmed diagnosis were eligible.

**Results:**

Fifteen studies met the inclusion criteria. Reported prevalence of human zTB ranged from 0% to 28%, with cattle and raw dairy the primary exposures. All confirmed zTB cases were *Mycobacterium bovis*. Eight of the 15 studies included livestock sampling, predominantly cattle and reported prevalence of zTB between 0% and 23%. Laboratory methods used included nearly a dozen different culture methods and a variety of molecular methods, some of which are not appropriate for zTB.

**Conclusions:**

This review revealed the need for appropriate and standardized laboratory diagnostic methods, and large prospective studies of at‐risk populations to determine exposures that lead to an increased risk of tuberculosis conversion/infection to better understand the true burden of disease.

Standardized, easy to implement laboratory diagnostics is an imperative focus for this scientific field to better identify the burden of zTB. Future studies pairing livestock and human subjects will allow better characterization of the high zTB transmission areas for targeted control and prevention programmes.

**Impacts**

- Despite the global focus on tuberculosis, the incidence and prevalence of zoonotic tuberculosis are still largely unknown, and likely underestimated.
- In many low‐ and middle‐income countries, deficiencies in laboratory capacity adversely impact the ability to correctly identify the disease‐causing species of tuberculosis, having negative impacts on both treatment success and future disease risk mitigation.
- Laboratory and surveillance capacity building are paramount to understanding the true burden of zoonotic tuberculosis and implementing appropriate disease control and prevention measures.

## 1 INTRODUCTION

Over the last two decades, zoonotic tuberculosis (zTB) has received increasing recognition as a growing public health threat, especially in low‐ and middle‐countries (LMICs) where the incidence of zTB is largely unknown. The *Mycobacterium tuberculosis* complex species that cause zTB include *M. bovis* (cattle), *M. caprae* (sheep and goats), *M. microti* (rodents), *M. mungi* (banded mongooses), *M. orygis* (members of the Bovidae family) and *M. pinnipedii* (seals and sea lions) (Jagielski et al., 2016). *M. bovis* is primarily transmitted to humans by cattle through the consumption of cattle products, such as unpasteurized milk and raw meat products contaminated with *M. bovis* lesions, and aerogenously (Center for Food Security & Public Health [CSFPH], 2009). Infection with or exposure to any *Mycobacterium* species can cause a positive reaction on the tuberculin skin test due to cross‐reactivity among the species but is not indicative of TB infection (Centers for Disease Control & Prevention [CDC], 2016). Clinically zTB cannot be distinguished from *M. tuberculosis*, although it may be more likely to cause extra‐pulmonary disease (World Health Organization [WHO], 2016).

Incidence estimates of zTB in LMICs are primarily based on a few studies of *M. bovis* and are not geographically representative as large‐scale human zTB incidence studies have not been undertaken and reported. In 2017, the WHO estimated the global incidence of zTB was 142,000 cases, out of the 10 million cases of tuberculosis, accounting for 1.4% of the global TB burden (WHO, 2016). The true incidence is likely underestimated due to poor surveillance programmes, under‐reporting and lack of laboratory confirmation of causative agent in LMICs where the most vulnerable populations reside (WHO, 2016). Direct smear microscopy, the most common diagnostic used in LMICs, does not differentiate between *Mycobacterium tuberculosis* complex species (Olea‐Popelka et al., 2017). Therefore, molecular diagnostics must be performed to speciate the bacteria. Of importance from a patient management perspective, *M. bovis* infections carry genes that make them intrinsically resistant to pyrazinamide, one of the drugs of choice in the standard first‐line anti‐TB treatment regimen, driving home the need to identify the species of tuberculosis at the time of diagnosis in order to successfully treat the patient (WHO, 2016).

To address prevention and control of human zTB, one must understand where it is naturally found. Cattle, buffalo and cervids are considered the maintenance hosts for *M. bovis* (CSFPH, 2009). The badger in the British Isles and the Australian brushtail possum are also maintenance hosts, which has complicated domestic livestock control programmes in the United Kingdom, The Republic of Ireland and New Zealand (Cousins, 2001). Spill‐over hosts include goats, sheep, pigs, dogs, cats, horses, many wild ruminants, camels and South American camelids among many others (CSFPH, 2009). One of these spill‐over hosts, the white‐tailed deer, continues to pose a threat to *M. bovis* eradication in a small portion of Michigan in the United States (CSFPH, 2009).

Although reporting is scarce, data from the OIE (2015) and from Müller et al.'s (2013) zTB review demonstrate that *M. bovis* has been documented in cattle and humans in every region of the world with higher prevalence reported or assumed in LMICs, making this a truly global public health problem (Cosivi et al., 1998; Müller et al., 2013; OIE, 2015). Out of 179 countries and territories reporting their bovine tuberculosis status during 2015 to 2016, more than half reported having the disease in wild and/or domestic animals (World Organization for Animal Health [OIE], WHO, Food and Agricultural Organization of the United Nations [FAO], 2015). In high‐income countries, cattle tuberculosis control programmes revolve around the test and slaughter technique (Cousins, 2001). This involves using either the intradermal tuberculin test or comparative intradermal tuberculin skin test (CIDT) to identify reactor animals, and segregating them for immediate slaughter and necropsy to identify lesions consistent with tuberculosis (Cousins, 2001; Wedlock, Skinner, de Lisle, & Buddle, 2002). CIDT, used primarily in LMICs to test cattle for bovine TB, involves injecting two sites, one with avian purified protein derivative (PPD) and the other with bovine PPD (Ameni et al., 2013). Many LMICs also have this type of programme, but they are often not implemented or enforced, nor adequately funded. Often times a more achievable programme in the beginning of disease eradication may be to test and segregate, which separates tuberculosis‐positive animals from those that are disease free without requiring immediate slaughter. This is logistically, economically and socially more feasible in many instances (Cousins, 2001; Wedlock et al., 2002).

Much of the literature documenting populations at increased risk of contracting *M. bovis* consists of retrospective data review and/or small sample sizes (Cordova et al., 2012; Haagsma, Tariq, Heederik, & Havelaar, 2012). Groups known to be at increased risk include animal husbandry workers, abattoir workers, dairy workers, live market workers, veterinary medicine personnel and HIV‐positive persons (Haagsma et al., 2012; Vayr et al., 2018). Populations that drink a great deal of raw milk without boiling it are also at higher risk of *M. bovis* infection (Silva et al., 2018). In much of Sub‐Saharan Africa, pastoralist communities live in close contact with their livestock year‐round, oftentimes keeping livestock in their home dwellings (Duguma, Abera, Zewdie, Belina, & Haro, 2017). Pastoralists and household members that live in close contact with their livestock are at increased risk of contracting *M. bovis*. Additionally, the commonplace practice of drinking raw milk, as indicated in one survey in Ethiopia that showed raw milk consumption at nearly 100% of the population, is another zTB risk factor for this population (Duguma et al., 2017).

The United Nations' Sustainable Development Goals (SDGs) include an emphasis on tuberculosis in subsection 3.3 of Goal Three which states, “By 2030, end the epidemics of AIDS, tuberculosis, malaria and neglected tropical diseases and combat hepatitis, water‐borne diseases and other communicable diseases” (United Nations [UN], 2015). The Stop TB Partnership, a unique international collaboration that operates through the UN's Office of Project Services (UNOPS), is actively working towards this SDG with an additional “90‐90‐90” target that by 2020 aims to diagnose and appropriately treat at least 90% of people with TB, with an emphasis on reaching at least 90% of the most vulnerable and at‐risk populations, and that at least 90% of TB patients treated have successful therapy outcomes (UNOPS, 2018). One vulnerable and at‐risk population are those that are HIV positive. In much of southern Africa, HIV and TB prevention and care must be considered in tandem due to the high rate of dual‐infection (World Health Organization [WHO], 2018). As LMICs make strides to control tuberculosis in humans, it is very likely that zTB will develop into a more significant public health threat because the prevention and control strategies used to address *M. tuberculosis*, which focus predominantly on human to human transmission, will have very little effect on the prevention and control of zTB, since the latter is primarily transmitted via food ingestion and animal handling. As part of the global effort to end the tuberculosis epidemic, it is important to understand the role zTB plays in the human tuberculosis burden, especially in LMICs with poor or no cattle tuberculosis control programmes. As part of the OIE's renewed emphasis on bovine TB and zTB documented in “Roadmap for Zoonotic Tuberculosis,” a challenging undertaking will be the effort to strengthen surveillance systems and expand the availability of “appropriate diagnostic tools” that can “identify and characterize zoonotic TB in people” (OIE, WHO, Food, & Agricultural Organization of the United Nations [FAO], 2017).

This systematic review aims to evaluate the quality of the literature that examines the association between human zTB in LMICs and frequent exposure to livestock and livestock products, and summarize current gaps in laboratory detection methods.

## 2 MATERIALS AND METHODS

### 2.1 Systematic review methodology

This systematic qualitative literature review follows the methodology published in The Navigation Guide and is described elsewhere (Woodruff & Sutton, 2014). This method is commonly used to evaluate environmental and occupational exposure risks, of which livestock is both, to support evidence‐based decision making, bridging the gap between clinical and environmental health. The Navigation Guide methodology follows PRISMA‐P (Preferred Reporting Items for Systematic review and Meta‐Analysis Protocols) guidelines and consists of specifying a study question, selecting the evidence and rating the quality and strength of the evidence (Woodruff & Sutton, 2014). PRISMA‐P guidelines were initially designed to guide rigorous and reliable systematic literature reviews that could develop healthcare practice guidelines and identify gaps in the literature to inform future research (Shamseer et al., 2015).

### 2.2 Study question

Our objective was to examine the association of human zoonotic tuberculosis in LMICs for people that have frequent exposure to livestock and/or livestock products compared to people with no or minimal exposure. Our PECO statement below describes the study question:

**Participants**: Humans living in low‐ and middle‐income countries.

**Exposure:** Livestock via husbandry, abattoir, markets, private use (any livestock used for food or fibre, such as wool or mohair), consumption of raw animal products or any other livestock or livestock product exposure not itemized.

**Comparator**: People that have no or minimal livestock exposure.

**Outcome**: Laboratory‐confirmed human zoonotic tuberculosis; culture or molecular diagnostics used to confirm a tuberculosis‐causing agent that is not *M. tuberculosis*.

### 2.3 Study selection

**Figure 1:**
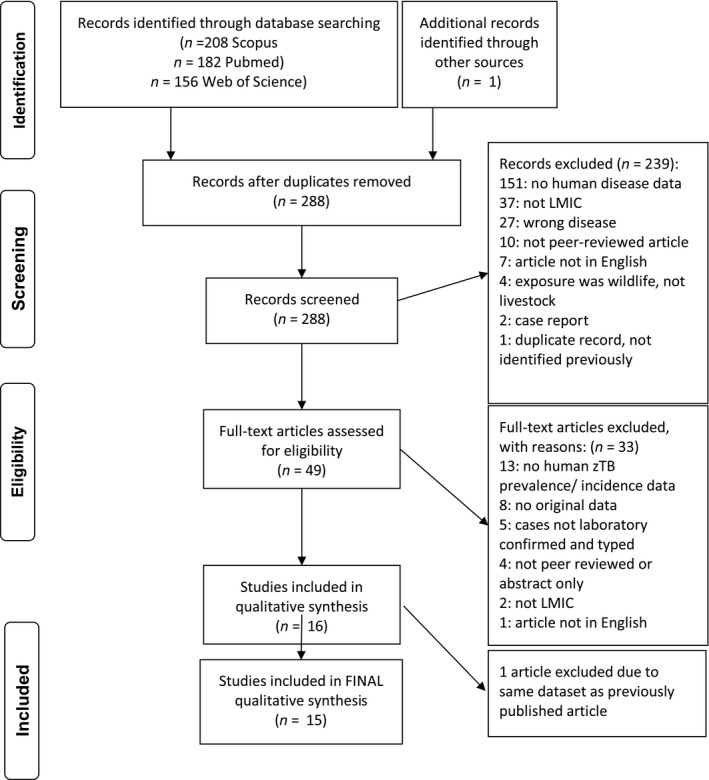
Study selection flowchart. Search terms for each database are provided in Table S1

Studies were selected among peer‐reviewed, scholarly journal articles in Scopus, PubMed via NCBI and Web of Science. Search terms, based on keywords from papers of interest, included: zoonotic AND tuberculosis AND livestock OR bovine OR cattle OR camel OR camelus OR human OR humans. Medical subject headings (MeSH) terms were used to search PubMed, and similar key words were used to search Scopus and Web of Science (Table S1). To include as many studies as possible, eligibility criteria were not limited by publication date or study type, with the exception of excluding evaluations of zTB interventions. Any article that met the following inclusion criteria published up to 11 October 2017 was included**:** published in English; in peer‐reviewed journals; included only populations living in LMICs; laboratory‐confirmed human zTB with speciation and; included exposure of interest which was contact with livestock via husbandry, abattoir, markets, private use (this includes any animal used for food or fibre), consumption of raw animal products or other close contact with livestock species. Case reports, data from high‐income countries, articles not published in English and articles not presenting original data were excluded. The Study Selection Flowchart (Figure 1) outlines records at each step of the screening process.

Search results were downloaded to RefWorks, which was used to remove duplicate records. All abstracts were exported from RefWorks to Excel, which was used to screen title and abstracts for the first‐round screening, followed by full‐text screening. Figure 1 shows the adaptation of the PRISMA study selection flowchart. Excel was used to capture data extraction and summarization. Key data extracted from each study are in Table 1. All data extracted from each selected study are available in Table S18.

**Table 1:** Summary of key study characteristics of zTB in human observational studies

Author** (**Publishing Year)	Objective of study	Assess livestock exposure/ raw dairy?	Location of Study (Country)	Human case inclusion	Study Design	Livestock testing?	Human Sample Size	% positive for zTB as total of all samples	% positive for zTB as total of all Mycobacterium positive samples
Ameni et al. (2013)	Investigate the transmission of MTC between cattle and their owners in Central Ethiopia	Yes	Ethiopia	Farmers: cases clinically diagnosed TB; controls did not have TB history in the last decade	Case‐control	Herds of AFB‐positive and TB‐free households CIDT tested; strong reactors slaughtered and necropsied; TB lesions cultured	257; 146 cases; 141 controls	0%	n/a
Gumi et al. (2012)	Assess presence of *M. bovis* among human TB patients; describe mycobacterial strains circulating in SE Ethiopian pastoralists and livestock	No	Ethiopia	Clinically diagnosed with pulmonary TB or TB lymphadenitis	Cross‐sectional	Cattle, goats, camels with suspected TB lesions at abattoirs	292	1.02% (3/292)	1.6% (3/183) AFB positive
Malama et al. (2014)	Molecular identification of *M. bovis* in humans and cattle; determine zoonotic significance in Namwala district of Zambia	Yes	Zambia	Clinically suspected of pulmonary TB	Cross‐sectional	Slaughtered cattle at 2 abattoirs	100	2% (2/100)	5.6% (2/36) total MTC
Mengistu, Enquselassie, Mulatu, Hailu, & Beyene, (2015)	Investigate/ determine the prevalence of BTB and see possible role of cattle in the epidemiology of human TB and isolate MTC species in the Wollo Zone, Amhara National Regional State	Yes	Ethiopia	Persons with chronic cough of 2 + weeks, owned cattle, not under treatment for TB, and > 15 years of age	Cross‐sectional	Cattle tested using CIDT, no further diagnostics for reactors	124	0%	n/a
Milian‐Suazo et al. (2010)	Compare spoligotypes from humans and cattle from the same geographic area to better understand the epidemiology of TB and the link between cattle and human cases of TB	Yes	Mexico	TB symptomatic patients, or dairy farm workers & local slaughterhouse workers	Cross‐sectional	Cattle from a local slaughterhouse with suspect lesions with cultured	552	6.2% (34/552)	n/a
Nuru et al. (2017)	Investigate the transmission of zTB between cattle and its owners in smallholder farms in northwestern Ethiopia	Yes	Ethiopia	Clinically diagnosed TBLN patients	Cross‐sectional	CIDT on cattle owned by TB patients and on TB‐free households	70	2.9% (2/70)	5% (2/40)
Prasad et al. (2005)	Utilize PCR‐RFLP and nested‐PCR to differentiate and detect *M. bovis* and *M. tuberculosis* and mixed infections in human and cattle extra‐pulmonary tuberculosis samples	No	India	Patients clinically suspected of TB	Cross‐sectional	Cattle clinically ill and clinically normal animals tested	331	10.3% (34/331); 8.7% (29/331) mixed *M. bovis* and *M. tb* infection	29.6% (34/115); 25.2% (29/115) mixed *M. bovis* and *M. tb* infection
Rahman et al. (2015)	Evaluate PCR‐based diagnostic test specific for *M. bovis* for testing bovine and human bio samples for bTB and to identify potential risk factors for its human transmission	Yes	Bangladesh	Chest radiograph and direct smear microscopy‐positive TB patients	Cross‐sectional	300 bovine milk samples: 200 from healthy animals and 100 from debilitated cows	90	6.7% (6/90)	n/a
Firdessa et al. (2013)	Explore public health risk for bovine TB in Ethiopia using molecular typing to characterize isolates from TBLN and pulmonary TB patients; define role of M. bovis in human TB	Yes	Ethiopia	Patients suspected of TBLN or pulmonary TB	Cross‐sectional	No	2,151	n/a	0.4% (4/964) AFB positive
Kazwala et al. (2001)	Determine the involvement of *M. bovis* in TB cases presenting at TB clinics in rural areas in the study area.	Yes‐ livestock keeping	Tanzania	Clinically diagnosed pulmonary or extra‐pulmonary TB	Cross‐sectional	No	149	4.7% (7/149)	15.9% (7/44) Mycobacteria culture positive
Khattak et al. (2016)	Determine the burden of active pulmonary TB caused by M. bovis in abattoir workers, butchers, veterinarians, livestock farmers and vet assistants and document associated risk factors	Yes	Pakistan	Government abattoir workers, butchers, farmers, vet assistants and veterinarians with chronic cough with sputum or blood	Cross‐sectional	No	103	4.9% (5/103)	n/a
Laniado‐Laborin et al. (2014)	Determine the prevalence of *M. bovis* human disease among patients referred to the Tuberculosis Laboratory of the Tijuana General Hospital in Baja California, Mexico and to characterize the clinical isolates molecularly	No	Mexico	Culture‐positive cases of TB	Cross‐sectional	No	2,699	1.0% (27/2699)	4.5% (27/600)
Oloya et al. (2008)	Isolate and characterize mycobacteria causing cervical lymphadenitis in patients in the transhumant areas of Karamoja, Uganda	No	Uganda	Diagnosed with cervical lymphadenitis	Cross‐sectional	No	43	7% (3/43)	12.5% (3/24)
Portillo‐Gomez and Sosa‐Iglesias (2011)	To identify isolates of *Mycobacterium bovis* in humans and cattle by PCR, and establish the clinical and epidemiological importance of ZTB in humans	Yes	Mexico	Clinically diagnosed pulmonary or extra‐pulmonary TB	Cross‐sectional	No	124	28% (35/124)	n/a
Viegas et al. (2015)	Explore the public health risk for bovine TB in Maputo, the capital of Mozambique, by characterizing the isolates from TBLN case during one year in the Pathology Service of Maputo Central Hospital	No	Mozambique	Clinically suspected TBLN	Cross‐sectional	No	110	0%	n/a

Abbreviations: AFB, acid‐fast bacilli; bTB, bovine tuberculosis; CIDT, comparative intradermal tuberculin test; M. tb, *M. tuberculosis*; MTC, *Mycobacterium tuberculosis* complex; PCR, polymerase chain reaction; PCR‐ RFLP, polymerase chain reaction ‐ restriction fragment length polymorphism.

### 2.4 Quality and bias assessment

The quality of evidence across studies was rated using the Navigation Guide's modification of the GRADE (Grading Recommendations Assessment, Development and Evaluation) approach, elaborated in Table 2. All observational studies start out as “moderate‐quality” (Woodruff & Sutton, 2014). Reasons to downgrade the evidence include: serious risk of bias, serious inconsistency between studies, serious indirectness, serious imprecision (due to small study size) and likely publication bias. The overall body of evidence was upgraded if the following were seen: large effect size, strong positive correlation between exposure and outcome, and if all plausible confounding would reduce a true effect or suggest a spurious effect when the actual results show no effect (Woodruff & Sutton, 2014).

**Table 2:** Summary of quality of evidence and strength of evidence evaluation criteria

Evaluation factors	Summary of criteria
Quality downgrading factors	
Risk of bias	Study limitations – a substantial risk of bias across body of evidence
Indirectness	Evidence was not directly comparable to the question of interest (i.e. population, exposure, comparator, outcome)
Inconsistency	Widely different estimates of effect in similar populations (heterogeneity or variability in results)
Imprecision	Studies had few participants and few events (wide confidence intervals)
Publication bias	Studies missing from body of evidence, resulting in an over or underestimate of true effects from exposure
Quality upgrading factors	
Large magnitude of effect	Upgraded if modelling suggested confounding alone unlikely to explain associations that were judged to be of large magnitude
Dose response	Upgraded if consistent relationship between dose and response in one or multiple studies, and/or dose response across studies
Confounding minimizes effect	Upgraded if consideration of all plausible residual confounders or biases would underestimate the effect or suggest a spurious effect when results show no effect
Strength considerations	
Quality	Overall quality rating of the body of evidence (from above)
Effect estimate	Direction of the relationship seen between exposure and outcome
Confidence	Confidence in the effect estimate and likelihood that new studies would change the conclusion
Other	Any additional aspects of the data that may influence certainty

**Figure 2:**
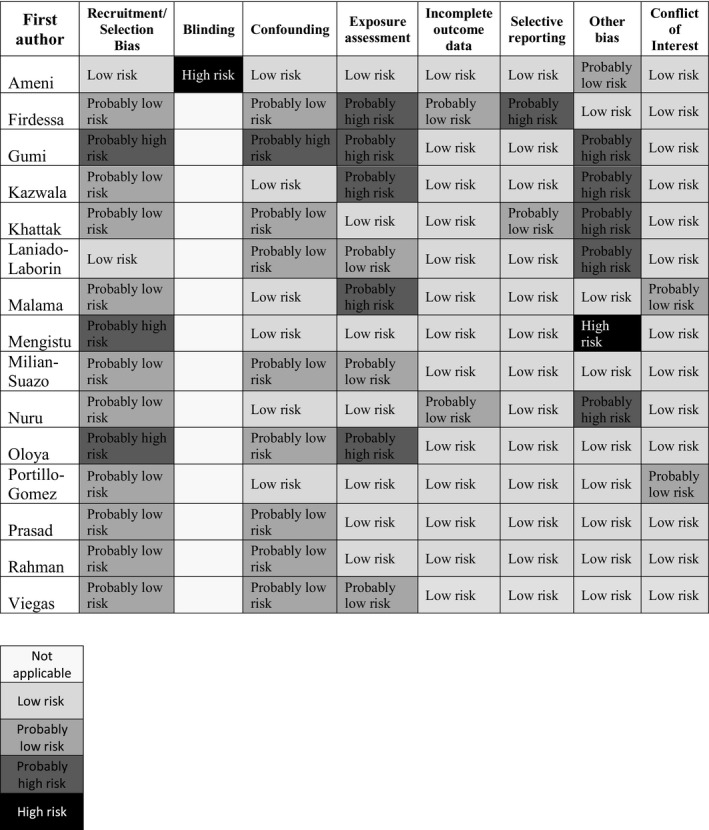
Summary of risk of bias judgements (low, probably low, probably high, high risk, not applicable) for each included study. Risk of bias designations is assigned according to reasons listed in Table S2, Characteristics of each study, Tables S3‐S17

Each study was assessed for risk of bias by evaluating the criteria found and reported in Figure 2. The following sources of bias were evaluated: recruitment strategy, exposure assessment, confounding, incomplete outcome data, reliability of laboratory results, selective reporting, conflict of interest or other possible biases. Although blinding is usually evaluated, it was not for this group of studies because the majority were of cross‐sectional design.

### 2.5 Rating the strength of evidence across studies

Rating the strength of the evidence across this body of literature allows us to summarize the evidence that will support or not support the association of human zTB and exposure to livestock and livestock products, and is based on the criteria from the Navigation Guide (see Table 2): (a) quality of the body of evidence, (b) direction of the effect estimate, (c) confidence in the effect estimate, meaning that a new study would not likely change our conclusion, and (d) any other compelling characteristics of the studies that might influence certainty (Johnson et al., 2014; Woodruff & Sutton, 2014). Strength of the evidence was determined by comparing the results of the four criteria listed with definitions for “sufficient evidence,” “limited evidence,” “inadequate evidence,” and evidence of lack of effect that can be found elsewhere (Woodruff & Sutton, 2014).

## 3 RESULTS

A total of 288 unique records were retrieved, and 15 met the inclusion criteria (Table 1). Reasons for exclusion at each level can be seen in Study Selection Flowchart, Figure 1. One article was included after manually searching references of included studies for pertinent articles that did not show up in our database searches. Although our search was not limited to *M. bovis*, this was the only zoonotic tuberculosis detected by the included studies. Other *Mycobacterium spp*. were detected by several of the excluded studies, but they were either human‐specific tuberculosis‐causing species or non‐tuberculous mycobacteria (NTM) species.

Studies were published between 2001 and 2017, in nine countries in Africa, Asia and Latin America (Table 1). The reported prevalence of *M. bovis* among tuberculosis‐suspect patients varies greatly among the included studies, from 0% to 28%. Four of six WHO regions were represented, with the majority of studies coming out of Africa (*n* = 9), followed by the Americas (*n* = 3), Southeast Asia (*n* = 2) and the Eastern Mediterranean region (*n* = 1). In all five studies from Ethiopia, the evaluated risk factor for zTB was living a pastoralist or smallholder farming lifestyle. These Ethiopian studies returned unexpectedly low reported prevalence of zTB, ranging from 0% to 2.9%, and two did not detect any *M. bovis* (the remaining three reported 0.4%–2.9%). In the three Mexican studies, the zTB reported prevalence ranged from 1.0% to 28%; primary risk factors identified included consumption of raw cheese and working with livestock. In a study in Tanzania, Kazwala et al. (2001) found a greater proportion of *M. bovis* in extra‐pulmonary cases (six out of seven cases) compared to a single pulmonary *M. bovis* tuberculosis case (X^2^ = 6.03; *df* = 1; *p* = .014).

All studies were cross‐sectional, with the exception of one case‐control study. Study sample size ranged from 43 to 2,699 and the majority were less than 200 (nine out of 15). Eight studies looked at *M. bovis* prevalence in cattle or other livestock in parallel with human subjects (Table 3). Of these eight studies, seven evaluated cattle, and one evaluated cattle, sheep, goats and camels.

**Table 3:** Summary table of livestock testing results for eight studies

First Author	Animal Species	Animal location at time of testing	Test methods and results					Study human subject zTB % (*M. bovis*)
			CIDT	Slaughter‐ TB lesions identified	Type of biologic sample	Culture	Molecular diagnostics *M. bovis* positive	
Ameni	Cattle	Household	36/ 2,033 (1.8%)	33/36 (91.7%)	Tissue	24/33 (72.7%)	3/24 (12.5%)	0%
Gumi	Cattle	Abattoirs	‐	50/ 5,250 (1.0%)	Tissue	36/50 (72%)	24/36 (66.7%)	1.02%
	Goats		‐	76/1744 (4.4%)	Tissue	9/76 (11.8%)	0 (0%)	
	Camels		‐	81/694 (11.7%)	Tissue	3/81 (3.7%)	0 (0%)	
Malama	Cattle	Abattoirs	‐	67/ 288 (23.3%)	Tissue	47/ 67 (70.1%)	25/ 47 (53.2%)	2%
Mengistu	Cattle	Household	5/ 381 (1.3%)	‐	‐	‐	‐	0%
Milian‐ Suazo	Cattle	Abattoir	‐	58/ NS	Tissue	NS	58/NS	6.2%
Nuru	Cattle	Household	0/NS	‐	‐	‐	‐	2.9%
Prasad	Cattle	Single government herd	‐	‐	Tissue	*M. tb*: 9/56 (16.1%)	*M. tb*: 16/56 (28.6%)	10.3%
					Tissue	*M. bovis*: 7/56 (12.5%)	*M. bovis*: 15/56 (26.8%)	
					Tissue	mixed: 1/56 (1.8%)	mixed: 20/56 (35.7%)	
Rahman	Cattle	Government and private dairy farms	‐	‐	Milk	‐	37/300	6.7%

Abbreviations: CIDT, comparative intradermal tuberculin test; *M. tb, M. tuberculosis*; NS, not stated; dashline (‐), test not performed.

Evaluation of the exposure of interest varied by study; some studies recorded exposure data for each subject, while other studies commented on general livestock exposure of the population from which study subjects were drawn. The Ethiopian case‐control study by Ameni et al. (2013) looked at the TB status of cattle in households of AFB‐positive patients, identified by both acid‐fast bacilli seen on smear and *Mycobacteria* culture, compared to cattle from households with no clinical history of TB in the last decade. Cattle that were owned by a TB case were much more likely to be positive on CIDT, with an odds ratio of 4.52 (95% CI 1.80, 11.36) (Ameni et al., 2013). This study noted not only zoonotic transmission of *M. bovis* but concern for a reverse zoonosis of *M. tuberculosis,* which is outside the scope of this review (Ameni et al., 2013). Other studies evaluated the following risk factors related to livestock: pastoralist lifestyle, presence of livestock in households, owning older compared to younger cattle, sharing the same “microenvironment” and watering points as livestock, drinking unboiled or raw milk, consumption of other raw dairy products (cheeses, yogurt), livestock blood consumption, duration of work in an abattoir as well as personal protective equipment used, eating raw meat, working on a dairy farm and husbandry condition of farmed cattle.

The occupational exposure assessment of zTB in Pakistan by Khattak, Mushtaq, Ahmad, Khan, and Haider (2016) found a statistically significant relationship between zTB occurrence in abattoir workers and longer duration of work; based on a bivariate frequency analysis, the chi‐square test gave a *p*‐value of < .05. However, no statistically significant relationship was seen when considering the type of animal work performed or education of the worker (Khattak et al., 2016). No other studies included in this review presented statistical analyses for zTB risk factors.

Table 4 summarizes the different laboratory detection methods utilized to identify *M. bovis* across this body of evidence. Eight different culturing techniques were utilized. Speciation was done by biochemical testing of the cultures and a variety of molecular diagnostic techniques. Three studies did DNA extraction without culture directly from the biological samples, and then performed PCR. Eight studies used spoligotyping, a series of PCR techniques, to identify and isolate the lineage of *M. bovis* for epidemiological tracing. Spoligotyping looks at the DNA polymorphism of the direct repeat (DR) region at one particular chromosomal locus, unique to the MTC bacteria, and then looks for different sequences known to *M. tuberculosis* or *M. bovis* (Milian‐Suazo, Perez‐Guerrero, Arriaga‐Diaz, & Escartin‐Chavez, 2010).

**Table 4:** Laboratory detection methods used for *M. bovis* by each study

First Author	Compliant with OIE culture recommendations				NOT compliant with OIE culture recommendations				Duration of culture (weeks)	Culture length OIE Compliant	PCR to detect MTC	PCR to detect *M. bovis*	Gene or Region of Difference targeted	Spoligotyping
	Lowenstein‐ Jensen + pyruvate media	Modified Middlebrook 7H11 or 7H10 media	Liquid media BBL mycobacteria growth indicator tube	Stonebrink + pyruvate media	LJ media	LJ + glycerol media	Stonebrink media	Coletsos media						
Ameni	x					x			5 – 8	N	x	x	RD 4, RD 9	x
Firdessa	x	x				x			8	Y	x	x	RD 4, RD 9	x
Gumi	x	x				x			8	Y	x	x	RD 4, RD 9	x
Kazwala	x					x			6+	N				
Khattak												x	NS	
Laniado‐Laborin			x		x		x		NS	UNK	x	x	RD1, RD 8	
Malama	x					x			8+	Y	x	x	RD 1, RD 4, RD 9, RD 12	x
Mengistu	x					x			12	Y	x	x	RD 4, RD 9	
Milian‐ Suazo				x	x				NS	UNK	x		MPB70	x
Nuru	x					x			8+	Y			NS	x
Oloya	x	x			x				12	Y	x		NS	x
Portillo‐Gomez	x					x			NS	UNK		x	oxyR	
Prasad											x	x	hupB	
Rahman					x			x	8	Y		x	NS	
Viegas			x		x		x		NS	UNK	x		IS6110	x

Abbreviations: LJ, Lowenstein Jensen; MTC, *Mycobacterium tuberculosis* complex; NS, not stated; N, no; PCR, polymerase chain reaction; RD, region of difference; UNK, unknown; x, indicates technique used; Y, yes.

### 3.1 Risk of bias

We concluded that there was generally “probably low risk” of bias across the 15 studies (Figure 2, Tables S2–S17). Areas with overall highest risk of bias were recruitment (or selection bias), exposure assessment and confounding. Several potential confounders for this particular study question are of note: occupation and duration, consumption of raw dairy or raw meat, age, TST (tuberculin skin test) status and education level. Three of the studies did not address any of these confounders because data were analysed based on microbiological diagnostics only. When confounders are not identified, they cannot be controlled for in the analysis, thereby potentially skewing conclusions drawn from the data. Detection bias was listed in “other bias” for several studies due to the laboratory techniques used or lack of description, discussed in greater detail in the discussion.

### 3.2 Quality of evidence

All of the studies aimed to detect tuberculosis, specifically *M. bovis* in human patients. However, not all studies evaluated individual risk factors for infection, and they instead considered risk factors for the population as a whole (Gumi et al., 2012; Nuru et al., 2017). Indirectness is a criterion of whether the article directly studies the outcome of interest, and it is not considered to be a downgrading factor for this body of evidence; all studies specifically reported on the outcome of interest, *M. bovis*, with mention of the exposure of interest, livestock. The extent to which livestock exposure is included in each study's analysis ranged from an individual subject exposure assessment as seen in Ameni et al. (2013) and others, to a generalization of the population's exposure as seen in the following two examples. In the Mozambican study by Viegas et al. (2015) in which isolates from tuberculous lymphadenitis (TBLN) cases were characterized to explore the public health risk of *M. bovis* in Maputo, no *M. bovis* was found in any human subjects, which were reported from urban and peri‐urban areas of Maputo, Mozambique where livestock and consumption of raw dairy were uncommon. In the Indian study by Prasad et al. (2005) that utilized PCR to detect *M. bovis* and *M. tuberculosis* in human and bovine extra‐pulmonary samples, individual human subjects' exposure to livestock was not directly reported.

Imprecision is considered to downgrade the body of evidence due to low prevalence and sample size; nine studies had 200 subjects or less. Inconsistency among the datasets does not contribute to downgrading of the evidence. Although the reported *M. bovis* prevalence varies among the studies, variation is expected. With an estimate of only 1.4% of global TB cases being attributed to zTB, having some studies report no cases, especially with small sample size, is expected. Comparing the data across studies is challenging because of differing case definitions and laboratory detection methods. Publication bias is possible considering the challenges in publishing data from LMICs but is not considered to play a significant role in the overall quality of the evidence presented.

Factors that would upgrade the quality of the evidence, such as large magnitude of effect and dose response, were not found to contribute to the overall quality. There was not enough evidence to suggest that confounding, such as patient demographics, would reduce the overall effect seen. However, if more studies included unique patient demographics that the authors believe could be confounders, as stated previously, the data may show different associations once these potential confounders are controlled. It is important to note that this review focuses on a very specific global population, whose description could itself be seen as potentially confounding such as education level, consumption of raw milk and regular exposure to livestock. In the future, more uniform and precise laboratory techniques may increase detection and therefore augment the effect seen. Overall this body of literature is rated as low quality due to the risk of biases and imprecision.

### 3.3 Strength of evidence

Our strength of the evidence considerations were as follows:

- Quality of body of evidence: Low.
- Direction of effect estimate: Increased risk of zTB with increased exposure to livestock and livestock products.
- Confidence in effect estimate: Unlikely that a new study would have an effect estimate that would show a different relationship.
- Other compelling attributes of the data that may influence certainty: None.

Based on the definitions for strength of evidence in the Johnson et al. (2014) article, we concluded that there was “limited” human evidence that livestock and raw livestock product exposure is associated with zTB in humans because when combining the results of all the studies, a “positive relationship is observed between exposure and outcome, where chance, bias and confounding cannot be ruled out with reasonable confidence” (Johnson et al., 2014). In addition, confidence in the relationship is constrained by factors such as “the number, size or quality of individual studies” described in the discussion below.

## 4 DISCUSSION

The results of this systematic review reveal gaps in current knowledge of zTB and limitations in the ability to detect it. Available peer‐reviewed literature on this subject is scarce considering the magnitude of this global public health concern. Many of the published studies have been limited by inadequate sample sizes and study designs. This review of laboratory methods used highlights a lack of laboratory detection technique standardization, which greatly affects data reliability and comparison.

The need for additional and larger studies is unquestionable. Only 33.3% (five out of 15) of included studies had more than 200 human subjects. With zTB accounting for an estimated 1.4% of the global TB burden, these sample sizes are simply not large enough for robust statistical analyses (WHO, 2016). Additionally, 13 of 15 studies recruited samples from patients that presented to healthcare facilities. This introduces substantial selection bias, especially among rural, poor populations that are less likely to seek care in a health institution. Another confounding dynamic within the human population is the HIV/AIDS epidemic that facilitates transmission and progression to active disease of any form of TB, with some studies showing a significantly increased proportion of *M. bovis* infections among HIV co‐infected TB patients compared with HIV‐negative TB patients (Müller et al., 2013). In the study out of Mozambique by Viegas et al. (2015), the majority of their subjects were HIV tested in tandem. While they did not detect any zTB in their population, 66.7% of their *M. tuberculosis*‐positive patients were HIV positive (Viegas et al., 2015). This clearly illustrates the need for larger sample sizes and more inclusive studies to better elucidate the zTB prevalence among differing global populations. Longitudinal studies that identify when seronegative individuals seroconvert to *M. bovis* would have better opportunities to elucidate the risk factors that led to exposure. Many of the studies conducted have relied on TST to identify when patients become tuberculosis positive, which can lead to the same reaction, regardless if the exposure was to a different type of Mycobacteria or NTM instead. Using the interferon‐gamma release assay (IGRA) blood test would allow positive identification of individuals upon infection with an MTC species (Hermansen, Thomsen, Lillebaek, & Ravn, 2014). Molecular diagnostics to identify the infection causing MTC species would then be performed. Large prospective cohort studies are needed to follow at‐risk populations over time with the goal of capturing tuberculosis conversion and/or onset of active zTB infection followed by immediate speciation of the causative agent.

There was a variety of laboratory diagnostic methods used to detect *M. bovis* (Table 4). In the 13 studies that cultured samples, eight different media and five different time periods were used. The OIE recommends solid egg‐based media, such as Lowenstein–Jensen [LJ], Coletsos base or Stonebrinks, and they recommend that these media contain either pyruvate or pyruvate and glycerol, the use of agar‐based media such as Middlebrook 7H10 or 7H11, or blood‐based agar media; cultures should be incubated for a minimum of eight weeks, although 10–12 weeks is recommended (OIE, 2015). Five studies used LJ media without pyruvate supplement, which means the media were not likely to grow *M. bovis*. Only four studies incubated cultures for longer than eight weeks. This shows the inadequate laboratory culture methods used, exemplifying detection bias within and among studies. Several of the studies used sputum as the diagnostic specimen, which will neglect extra‐pulmonary cases of tuberculosis.

Molecular diagnostic techniques varied among the studies as well. Some studies used PCR to detect MTC, then PCR for *M. bovis*, followed by spoligotyping. Other studies used spoligotyping after identifying a positive MTC on PCR. A variety of PCR techniques were used including PCR with gel electrophoresis, real‐time PCR and nested‐PCR. While most studies identified target genes (Table 4), others gave primer sequences only, or referenced previously described work, but did not detail their target genes (Khattak et al., 2016; Nuru et al., 2017; Oloya et al., 2008; Rahman et al., 2015). Comparing molecular techniques to culture results gave divergent results even within studies. In two Ethiopian studies that looked at livestock owners, only culture‐positive samples were subjected to molecular diagnostics (Ameni et al., 2013; Gumi et al., 2012). As is demonstrated in Table 4, culture‐positive samples had molecular detection rates of 0%–66.7%. In the Indian study by Prasad et al. (2005) that looked at human and cattle extra‐pulmonary lesions, each sample underwent culture and molecular diagnostics. Detection rates were not congruent. All of the diagnostic issues together likely lead to under‐diagnosis and misdiagnosis of *M. bovis* in people. In “The Roadmap for Zoonotic Tuberculosis,” the OIE calls for new, rapid diagnostic testing for the detection of *M. bovis* by 2025 (OIE, WHO, FAO, 2017). This goal is achievable with the collaboration of those currently using and working on molecular diagnostic testing. A standard, timely detection method for zTB is urgently needed.

This review has several strengths. It is the first one to our knowledge that specifically looks at global human zTB in relation to livestock exposure and does an in‐depth assessment of laboratory confirmatory methods. The most recent review on the topic of zTB was published in 2013 by Muller et al, and included studies published through 2010, with the aim to describe the occurrence of zTB globally. By focusing on LMICs, livestock exposure as a risk factor, and laboratory diagnostics, we have identified key gaps in available research, specifically among the world's most at‐risk populations. Cosivi et al. described zTB in the developing world in a WHO review in 1998 that emphasized the inadequacy of available data and the vulnerability of LMIC populations. Twenty years later, these same considerations are echoed in our findings. The results presented here can help shape future studies, particularly guiding better study design and laboratory method selection, and influence tuberculosis control and prevention policies at the local, regional and national levels.

This review also highlights the need for public health emphasis on global pasteurization, or at the very least, widespread boiling of milk. In the 2015 Bangladeshi article that analysed human and bovine samples (Rahman et al., 2015), they found that out of 300 milk samples, 12.3% (37 out of 300) were found to contain *M. bovis* by both PCR and culture (Table 3). The authors found that poor farm biosecurity and poor nutrition leading to ill health of the cattle were contributing management risk factors (Rahman et al., 2015). Consumption of raw milk by people is more likely to progress to extra‐pulmonary TB lesions (Melini, Melini, Luziatelli, & Ruzzi, 2017). Pasteurization completely removes *M. bovis* from dairy, having been designed to specifically destroy *M. tuberculosis* and *C. burnetti* (Melini et al., 2017). Increased uptake of dairy pasteurization in LMICs will remove a significant risk factor for zTB infection as well as several other pathogens such as *Brucella, Campylobacter, E. coli, Listeria, Staphylococcus, Streptococcus* and *Yersinia* (Melini et al., 2017).

There are several limitations to this systematic review. First, the exclusion of articles not written in English likely overlooked significant studies. Future iterations of this review should include articles in other languages. Secondly, reporting bias plays a role in the available data on zTB, particularly in LMICs where healthcare infrastructure is suboptimal and funding for studies is inadequate. Selection bias is another big limitation, as many infected people were likely excluded because they did not report to a modern healthcare facility for care. Detection bias is very likely as only laboratory‐confirmed cases were included. In many developing countries, the only laboratory diagnostic performed on TB suspect patients is direct smear microscopy, which will not identify the causative species, further limiting the available diagnostic data that can be collected. The previously discussed lack of standardization of laboratory methods limits the reliability of and comparability of results across studies. Furthermore, the cases reported by these studies are not representative of any population other than their study population; country, regional or global prevalence cannot be inferred from this information. The scarcity of articles on our topic is another indication of the lack of importance placed on the study of exposure and infection with zTB by the global health community.

## 5 CONCLUSION

The body of scientific evidence presented here, although the studies are of overall low quality, shows that there is likely a relationship between livestock and livestock product exposure and human zTB in LMICs. The first step in better defining the burden of zTB is vastly improved livestock surveillance, both in herds and at slaughter. This will identify locations in which human zTB is most likely to occur. Efforts can then be focused on those at‐risk human populations with an emphasis on appropriate laboratory methods to properly diagnose, and therefore treat, zTB cases.

Future large‐scale prospective studies should pair human and livestock data to better define the location, extent and transmission patterns of zTB, which will energize LMIC governments and development agencies to allocate resources to improving livestock tuberculosis surveillance and control programmes and more widespread uptake of dairy pasteurization. With global focus on the UN's SDGs, there is anticipation that additional resources will be available to study, quantify and help prevent zTB, as part of the focus to attain SDG 3.3 and Stop TB Partnership's 90‐90‐90 goal of ending the global TB epidemic (UN, 2015; UNOPS, 2018).

## CONFLICT OF INTEREST

The views presented in this paper are those of the authors and do not necessarily reflect the views or policies of the George Washington University. The authors declare no competing financial interests or other source of conflict of interest.

## ETHICAL CONSIDERATIONS

As this work was a systematic literature review, no human or animal subjects were directly involved in this literature review.
