# Supplementary material for: Human zoonotic tuberculosis and livestock exposure in low‐ and middle‐income countries: A systematic review identifying challenges in laboratory diagnosis
Source: Zoonoses Public Health. 2020 Jan 9;67(2):97–111. doi: 10.1111/zph.12684 (PMC7027859; doi:10.1111/zph.12684)
Supplement: Supplementary file 1 [file ZPH-67-97-s001.docx]

Supplemental Material

Human Zoonotic Tuberculosis and Livestock Exposure in Low- and Middle-Income Countries: A Systematic Review Identifying Challenges in Laboratory Diagnosis

Sarah Anne Luciano, Amira Roess

Table of Contents Page

**Table S1.** Search terms used by database for systematic literature search. 2

**Table S2.** Summary of risk of bias designations for individual human studies. 3

**Table S3**. Characteristics of Ameni et al. 2013. 4

**Table S4.** Characteristics of Firdessa et al. 2013. 5

**Table S5.** Characteristics of Gumi et al. 2012. 6

**Table S6.** Characteristics of Kazwala et al. 2001. 7

**Table S7.** Characteristics of Khattak et al. 2016. 8

**Table S8.** Characteristics of Laniado-Laborin et al. 2001. 9

**Table S9.** Characteristics of Malama et al. 2014. 10

**Table S10.** Characteristics of Mengistu et al. 2015. 11

**Table S11.** Characteristics of Milian-Suazo et al. 2010. 12

**Table S12.** Characteristics of Nuru et al. 2017. 13

**Table S13.** Characteristics of Oloya et al. 2008. 14

**Table S14.** Characteristics of Portillo-Gomez and Sosa-Iglesias 2011. 15

**Table S15.** Characteristics of Prasad et al. 2005. 16

**Table S16.** Characteristics of Rahman et al. 2015. 17

**Table S17.** Characteristics of Viegas et al. 2015. 18

**Table S18**. Data extraction from 15 included studies. 19

**Table S1.** Search terms used by database for systematic literature search.

| **Database searched** | **Search terms used** |
| --- | --- |
| PubMed | (((((("livestock"[MeSH Terms] OR "livestock"[All Fields]) OR ("cattle"[MeSH Terms] OR "cattle"[All Fields])) OR ("cattle"[MeSH Terms] OR "cattle"[All Fields] OR "bovine"[All Fields])) OR ("camelus"[MeSH Terms] OR "camelus"[All Fields] OR "camel"[All Fields])) OR ("camelus"[MeSH Terms] OR "camelus"[All Fields])) AND (zoonotic[All Fields] AND ("tuberculosis"[MeSH Terms] OR "tuberculosis"[All Fields]))) AND ("humans"[MeSH Terms] OR "humans"[All Fields] OR "human"[All Fields]) |
| Web of Science | **TOPIC:**((livestock OR cattle OR bovine OR camel OR camelus)) *AND* **TOPIC:**(zoonotic tuberculosis) *AND* **TOPIC:**(human) |
| Scopus | ( TITLE-ABS-KEY ( livestock )  OR  TITLE-ABS-KEY ( cattle )  OR  TITLE-ABS-KEY ( bovine )  OR  TITLE-ABS-KEY ( camel )  OR  TITLE-ABS-KEY ( camelus )  AND  TITLE-ABS-KEY ( zoonotic  AND tuberculosis )  AND  TITLE-ABS-KEY ( human ) ) |

**Table S2.** Summary of risk of bias designations for individual human studies.

| **Risk of bias domains** | **Low risk of bias designation** |
| --- | --- |
| Recruitment strategy | Participant recruitment protects against selection bias |
| Blinding | Knowledge of exposure is prevented when assessing outcome; generally not applicable for cross-sectional observational studies |
| Exposure assessment | Risk of exposure misclassification is minimized through validated methods |
| Confounding | Important potential confounders were appropriately accounted for |
| Incomplete outcome data | Any missing outcome data is not likely to introduce bias |
| Selective outcome reporting | All outcomes specified in methods have been reported |
| Conflict of interest | Study free of support from individual or entity having financial interest in outcome of study |
| Other bias | Study appears to be free of other sources of bias |

| **Table S3.** Characteristics of Ameni et al. 2013. | |  |  |  |  |  |  |
| --- | --- | --- | --- | --- | --- | --- | --- |
| **Bias** | **Rating** | **Support for judgement** |  |  |  |  |  |
| Recruitment | Low risk | Recruited both cases and controls; strategy was well defined and consistent |  |  |  |  |  |
| Blinding | High risk | Authors did not address blinding at any level: interview, laboratory, human or animal. |  |  |  |  |  |
| Confounding | Low risk | Analyzed the data for various confounders: gender, age, chewing tobacco use, breed of cattle owned, TB contact, etc. |  |  |  |  |  |
| Exposure assessment | Low risk | Risk factors as above were analyzed to include consumption of raw milk as well |  |  |  |  |  |
| Incomplete outcome data | Low risk | No indication of incomplete data sets |  |  |  |  |  |
| Selective reporting | Low risk | No indication of selective reporting |  |  |  |  |  |
| Other bias | Probably low risk | Detection bias: culture methods did not include culture on modified Middlebrook 7H11 medium optimized for *M. bovis* |  |  |  |  |  |
| Conflict of Interest | Low risk | Funding and affiliations were government and academia |  |  |  |  |  |
|  |  |  |  |  |  |  |  |
| Design: | Case-control |  |  |  |  |  |  |
| Participants: | Farmers living in Central Ethiopia; cases presented to area health institutions;  controls did not have TB history in the last decade | | | | | | |
| Exposure: | Cattle rearing |  |  |  |  |  |  |
| Outcome: | Clinically suspected TB; cultured and typed in lab | |  |  |  |  |  |
| Notes: | Great study design, and discussed improvements for future studies | |  |  |  |  |  |

| **Table S4.** Characteristics of Firdessa et al. 2013. | |  |  |  |  |
| --- | --- | --- | --- | --- | --- |
| **Bias** | **Rating** | **Support for judgement** |  |  |  |
| Recruitment | Probably low risk | Large sample size recruited over a large study area |  |  |  |
| Blinding | Not applicable |  |  |  |  |
| Confounding | Probably low risk | Overall demographics and risk factors discussed for the community, not individual patients |  |  |  |
| Exposure assessment | Probably high risk | Individual risk factors were not reported beyond "pastoralist community" for some of the samples |  |  |  |
| Incomplete outcome data | Probably low risk | Outcomes discussed by authors were reported, however, data was reported based off the total AFB positive samples instead of the study total samples |  |  |  |
| Selective reporting | Probably high risk | Total samples collected and analyzed not reported; focused on 964 culture positive samples without specifying number of culture negatives |  |  |  |
| Other bias | Low risk | No other biases were suspected |  |  |  |
| Conflict of Interest | Low risk | All funding and affiliations appear to be from government and academia |  |  |  |
|  |  |  |  |  |  |
| Design: | Cross-sectional |  |  |  |  |
| Participants: | Patients suspected of TBLN or pul TB between 2006-2010 seen at hospitals in | | |  |  |
|  | Gondar, Woldiya, Ghimbi, Butajira, Negelle, Fiche, Jinka, Filtu and Addis Ababa | | |  |  |
| Exposure: | Pastoralist lifestyle |  |  |  |  |
| Outcome: | TBLN or pul TB; laboratory isolated and typed | |  |  |  |
| Notes: | Samples were cultured prior to molecular diagnostics | |  |  |  |

|  |  |  | |  |  | |  | | |  |  |  |
| --- | --- | --- | --- | --- | --- | --- | --- | --- | --- | --- | --- | --- |
| **Table S5.** Characteristics of Gumi et al. 2012. | | |  | | |  | |  |  | |  |  |
| **Bias** | **Rating** | | **Support for judgement** | | |  | |  |  | |  |  |
| Recruitment | Probably high risk | | Enrolled clinically diagnosed patients from 2 area hospitals; did not state enrollment percentage; in a pastoralist community a high percentage of the ill population may not seek institutional care | | |  | |  |  | |  |  |
| Blinding | Not applicable | |  | | |  | |  |  | |  |  |
| Confounding | Probably high risk | | Individual demographics and risk factors not considered in analysis | | |  | |  |  | |  |  |
| Exposure assessment | Probably high risk | | Individual exposure assessment not documented; pastoralist lifestyle assumed for all enrolled | | |  | |  |  | |  |  |
| Incomplete outcome data | Low risk | | No incomplete data set is indicated; results reported for all samples | | |  | |  |  | |  |  |
| Selective reporting | Low risk | | No indication of selective reporting. | | |  | |  |  | |  |  |
| Other bias | Probably high risk | | Detection bias considered as the methods section does not indicate use of positive and negative controls at each step. | | |  | |  |  | |  |  |
| Conflict of Interest | Low risk | | Funding through government and academic institutions | | |  | |  |  | |  |  |
|  |  | |  | | |  | |  |  | |  |  |
| Design: | Cross-sectional | |  | | |  | |  |  | |  |  |
| Participants: | Patients clinically diagnosed with pulmonary TB or TB lymphadenitis in Negelle and Filtu hospitals Mar 2008 and Feb 2010 | | | | | | | | | | | |
|  | Cattle, goats, camels w/ suspected TB lesions at Negelle, Filtu, Mojo and Addis Ababa abattoirs | | | | | | | | | |  |  |
| Exposure: | Pastoralist lifestyle | |  | | |  | |  |  | |  |  |
| Outcome: | Clinical TB, laboratory confirmed | | | | |  | |  |  | |  |  |
| Notes: | Laboratory methods write up was weak | | | | |  | |  |  | |  |  |

| **Table S6.** Characteristics of Kazwala et al. 2001. | |  |  |  |  |  |  |
| --- | --- | --- | --- | --- | --- | --- | --- |
| **Bias** | **Rating** | **Support for judgement** |  |  |  |  |  |
| Recruitment | Probably low risk | Patients seen at various medical facilities for clinical TB were enrolled, authors do not indicate proportion of patients vs those actually enrolled. |  |  |  |  |  |
| Blinding | Not applicable |  |  |  |  |  |  |
| Confounding | Low risk | Samples were analyzed for molecular diagnostics only; no patient demographic data was associated |  |  |  |  |  |
| Exposure assessment | Probably high risk | Individual patient exposure was not assessed; overall community livestock keeping and risk factors were discussed. |  |  |  |  |  |
| Incomplete outcome data | Low risk | All data outcomes discussed were reported. |  |  |  |  |  |
| Selective reporting | Low risk | No indication of selective data reporting. |  |  |  |  |  |
| Other bias | Probably high risk | Detection bias: Culture methods and Mycobacterium typing methods used were not ideal; likely underestimated outcome occurrence |  |  |  |  |  |
| Conflict of Interest | Low risk | Funding was through the British Department of International Development |  |  |  |  |  |
|  |  |  |  |  |  |  |  |
| Design: | Cross-sectional |  |  |  |  |  |  |
| Participants: | TB patients: three hospitals in Arusha region in the North, 3 hospitals and 2 health centers in the Southern Highlands, Tanzania | | | | | | |
| Exposure: | Living in a rural area |  |  |  |  |  |  |
| Outcome: | Clinical TB; laboratory typed Mycobacterium | |  |  |  |  |  |
| Notes: | Did not use PCR to type, used visual and biochemical methods | |  |  |  |  |  |

| \| **Table S7.** Characteristics of Khattak et al. 2016. \| \|  \|  \|  \|  \|  \| \| --- \| --- \| --- \| --- \| --- \| --- \| --- \| \| **Bias** \| **Rating** \| **Support for judgement** \|  \|  \|  \|  \| \| Recruitment \| Probably low risk \| Recruited among all abattoir workers, vet assistants, veterinarians, butchers and a convenience sample of livestock farmers with chronic cough \|  \|  \|  \|  \| \| Blinding \| Not applicable \|  \|  \|  \|  \|  \| \| Confounding \| Probably low risk \| For abattoir workers, analyzed data by age, education, duration of work and type of work \|  \|  \|  \|  \| \| Exposure assessment \| Low risk \| In depth questionnaires administered to determine type of work, PPE used, etc \|  \|  \|  \|  \| \| Incomplete outcome data \| Low risk \| All data outcomes discussed were reported \|  \|  \|  \|  \| \| Selective reporting \| Probably low risk \| Insignificant p-values were not reported \|  \|  \|  \|  \| \| Other bias \| Probably high risk \| Detection bias: Authors addressed possibility of detecting more cases if they had included TBLN and latent infections \|  \|  \|  \|  \| \| Conflict of Interest \| Low risk \| Funding through Pakistani government \|  \|  \|  \|  \| \|  \|  \|  \|  \|  \|  \|  \| \| Design: \| Cross-sectional \|  \|  \|  \|  \|  \| \| Participants: \| Symptomatic abattoir workers, butchers, veterinarians, livestock farmers and vet assistants in Peshwar district \| \| \| \| \| \| \| Exposure: \| Livestock \|  \|  \|  \|  \|  \| \| Outcome: \| Pulmonary TB; laboratory confirmation of *M. bovis* \| \|  \|  \|  \|  \| |  |  |  |  |  |  |  |
| --- | --- | --- | --- | --- | --- | --- | --- | --- | --- | --- | --- | --- | --- | --- | --- | --- | --- | --- | --- | --- | --- | --- | --- | --- | --- | --- | --- | --- | --- | --- | --- | --- | --- | --- | --- | --- | --- | --- | --- | --- | --- | --- | --- | --- | --- | --- | --- | --- | --- | --- | --- | --- | --- | --- | --- | --- | --- | --- | --- | --- | --- | --- | --- | --- | --- | --- | --- | --- | --- | --- | --- | --- | --- | --- | --- | --- | --- | --- | --- | --- | --- | --- | --- | --- | --- | --- | --- | --- | --- | --- | --- | --- | --- | --- | --- | --- | --- | --- | --- | --- | --- | --- | --- | --- | --- | --- | --- | --- | --- | --- | --- | --- |

| **Table S8.** Characteristics of Laniado-Laborin et al. 2001. | | |  |  |  |
| --- | --- | --- | --- | --- | --- |
| **Bias** | **Rating** | **Support for judgement** |  |  |  |
| Recruitment | Low risk | All culture proven TB cases reported by the TB Lab of the Tijuana General hospital were included. |  |  |  |
| Blinding | Not applicable |  |  |  |  |
| Confounding | Probably low risk | Samples were analyzed for molecular diagnostics only; patient demographic data associated was minimal and not used in the analysis |  |  |  |
| Exposure assessment | Probably low risk | Individual data was not presented; but authors discussed overall demographics and risk factors |  |  |  |
| Incomplete outcome data | Low risk | All data outcomes mentioned were discussed. |  |  |  |
| Selective reporting | Low risk | No evidence of selective reporting |  |  |  |
| Other bias | Probably high risk | Detection bias- optimized culture media not used for the first years of the study; only 25% of new TB cases were submitted for culture |  |  |  |
| Conflict of Interest | Low risk | Affiliations and funding are through the hospital and academic institutions |  |  |  |
|  |  |  |  |  |  |
| Design: | Cross-sectional |  |  |  |  |
| Participants: | All culture proven cases of TB reported by the Tuberculosis Laboratory of the Tijuana General Hospital | | | | |
| Exposure: | Consumption of raw dairy | |  |  |  |
| Outcome: | Clinically diagnosed TB, confirmed and typed in the lab | |  |  |  |
| Notes: | Identified strains initially by drug resistance testing; if resistant to Z, used PCR to see if *M. bovis* | | | | |
|  |  |  |  |  |  |

| **Table S9.** Characteristics of Malama et al. 2014. | |  |  | |  |  |
| --- | --- | --- | --- | --- | --- | --- |
| **Bias** | **Rating** | **Support for judgement** |  | |  |  |
| Recruitment | Probably Low risk | Recruited patients from 3 medical facilities based on clinical symptoms of pulmonary TB; only patients with viable samples were included |  | |  |  |
| Blinding | Not applicable |  |  | |  |  |
| Confounding | Low risk | Samples were analyzed for molecular diagnostics only; no patient demographic data was associated |  | |  |  |
| Exposure assessment | Probably high risk | No individual patient risk factors were annotated, only the general demographics and livestock keeping of the study area |  | |  |  |
| Incomplete outcome data | Low risk | All stated outcomes were reported. |  | |  |  |
| Selective reporting | Low risk | No indication of selective reporting |  | |  |  |
| Other bias | Low risk | No other biases are suspected. |  | |  |  |
| Conflict of Interest | Probably Low risk | Funding not directly stated; Authors' affiliations include government and academia of Zambia, Norway and the UK |  | |  |  |
|  |  |  |  | |  |  |
| Design: | Cross-sectional |  |  | |  |  |
| Participants: | Patients suspected of pulmonary TB seeking care at 3 medical facilities from April 2011 to July 2012 | | | | | |
| Exposure: | Agricultural economy; majority of people own livestock and drink raw milk | | |  |  |  |
| Outcome: | Pulmonary TB; laboratory confirmed and typed | |  | |  |  |
| Notes: | Discussed concern of human to human transmission of *M. bovis* | |  | |  |  |

| **Table S10.** Characteristics of Mengistu et al. 2015. | | |  |  |  |  |  |
| --- | --- | --- | --- | --- | --- | --- | --- |
| **Bias** | **Rating** | **Support for judgement** |  |  |  |  |  |
| Recruitment | Probably high risk | Community based enrollment of suspected TB cases, did not state method of recruitment or percent approached vs enrolled. |  |  |  |  |  |
| Blinding | Not applicable |  |  |  |  |  |  |
| Confounding | Low risk | Various demographics and risk factors were collected and used in the analysis. |  |  |  |  |  |
| Exposure assessment | Low risk | Subjects enrolled only if they owned cattle; various risk factors were documented for each subject |  |  |  |  |  |
| Incomplete outcome data | Low risk | All outcomes discussed were reported. |  |  |  |  |  |
| Selective reporting | Low risk | No evidence of selective reporting |  |  |  |  |  |
| Other bias | High risk | Detection bias: laboratory methods described used media not optimized for *M bovis* growth, and included "subculturing" in undisclosed media type |  |  |  |  |  |
| Conflict of Interest | Low risk | Funding and affiliations were academic and research institutions |  |  |  |  |  |
|  |  |  |  |  |  |  |  |
| Design: | Cross-sectional, community based | |  |  |  |  |  |
| Participants: | Persons in study areas with chronic cough 2+ weeks, owned cattle, not under treatment for TB, > 15 yrs old | | | | | | |
| Exposure: | Owning cattle |  |  |  |  |  |  |
| Outcome: | Pulmonary TB, laboratory confirmed | |  |  |  |  |  |
| Notes: | No individuals were screened at a medical facility prior to enrollment | |  |  |  |  |  |

| **Table S11.** Characteristics of Milian-Suazo et al. 2010. | | |  |  |  |  |  |
| --- | --- | --- | --- | --- | --- | --- | --- |
| **Bias** | **Rating** | **Support for judgement** |  |  |  |  |  |
| Recruitment | Probably Low risk | Convenience sampling used, but from 3 populations: TB symptomatic patients, dairy farm workers and slaughterhouse workers |  |  |  |  |  |
| Blinding | Not applicable |  |  |  |  |  |  |
| Confounding | Probably Low risk | Samples were molecularly analyzed, with the focus on molecular diagnostics, not sample origin; demographics presented were predominantly occupation only |  |  |  |  |  |
| Exposure assessment | Probably Low risk | Exposure assessment was straightforward for two populations, but not for the TB symptomatic patients. |  |  |  |  |  |
| Incomplete outcome data | Low risk | All data outcomes discussed were presented. |  |  |  |  |  |
| Selective reporting | Low risk | No evidence of selective reporting |  |  |  |  |  |
| Other bias | Low risk | No other biases are suspected. |  |  |  |  |  |
| Conflict of Interest | Low risk | Funding and affiliations were disclosed: Fondos Sectoriales SALUD-CONACYT, Mexico |  |  |  |  |  |
|  |  |  |  |  |  |  |  |
| Design: | Cross-sectional |  |  |  |  |  |  |
| Participants: | TB symptomatic patients sent to diagnostic lab; dairy farm and slaughterhouse workers in Queretero, Mexico | | | | | | |
| Exposure: | High-risk occupation, consuming unpasteurized dairy | |  |  |  |  |  |
| Outcome: | TB; confirmed and typed in the lab | |  |  |  |  |  |
| Notes: | Animal samples taken at slaughter to compare epidemiologic spoligotypes | |  |  |  |  |  |

| **Table S12.** Characteristics of Nuru et al. 2017. | |  |  |  |  |  |  |
| --- | --- | --- | --- | --- | --- | --- | --- |
| **Bias** | **Rating** | **Support for judgement** |  |  |  |  |  |
| Recruitment | Probably Low risk | Does not state what percentage of TBLN patients were recruited or over what timeframe |  |  |  |  |  |
| Blinding | Not applicable |  |  |  |  |  |  |
| Confounding | Low risk | Several confounders were addressed to include age, gender, education, consumption of raw dairy, undercooked meat |  |  |  |  |  |
| Exposure assessment | Low risk | All participants were smallholder farmers owning livestock |  |  |  |  |  |
| Incomplete outcome data | Probably Low risk | All human data outcome were reported; sample size of cattle population was not reported |  |  |  |  |  |
| Selective reporting | Low risk | No indication that any data sets were withheld. |  |  |  |  |  |
| Other bias | Probably high risk | Detection bias is suspected due to culture techniques utilized. |  |  |  |  |  |
| Conflict of Interest | Low risk | All funding sources and affiliations were academic or governmental. |  |  |  |  |  |
|  |  |  |  |  |  |  |  |
| Design: | Cross-sectional |  |  |  |  |  |  |
| Participants: | Clinically diagnosed TBLN patients seen at the Felegehiwot Comprehensive Specialized Hospital, Bahir Dar City | | | | | |  |
| Exposure: | Smallholder farmer owning livestock | |  |  |  |  |  |
| Outcome: | Clinical diagnosed TBLN, confirmed by lab | |  |  |  |  |  |
| Notes: | Also did CIDT on undisclosed number of cattle in TB and TB free households; all negative | | | |  |  |  |

| **Table S13.** Characteristics of Oloya et al. 2008. | |  |  |  |  |
| --- | --- | --- | --- | --- | --- |
| **Bias** | **Rating** | **Support for judgement** |  |  |  |
| Recruitment | Probably high risk | Only persons that reported to 2 area hospitals for cervical lymphadenitis were included; in a pastoralist community a high percentage of the ill population may not seek institutional care |  |  |  |
| Blinding | Not applicable |  |  |  |  |
| Confounding | Probably Low risk | Age, gender and occupation were taken into account |  |  |  |
| Exposure assessment | Probably high risk | Individual patients were not interviewed for extensive exposure assessment |  |  |  |
| Incomplete outcome data | Low risk | Incomplete outcome data was not present |  |  |  |
| Selective reporting | Low risk | The study does not suggest any selective reporting |  |  |  |
| Other bias | Low risk | No other potential biases are suspected |  |  |  |
| Conflict of Interest | Low risk | Authors report no conflict of interest; funding is from government or academia only |  |  |  |
|  |  |  |  |  |  |
| Design: | Cross-sectional |  |  |  |  |
| Participants: | Patients reporting to the TB units of the Moroto and Matany hospitals for cervical lymphadenitis | | | | |
| Exposure: | Pastoral lifestyle; livestock, raw dairy products | |  |  |  |
| Outcome: | Cervical lymphadenitis | |  |  |  |
| Notes: | MTC and MAC was evaluated | |  |  |  |

| **Table S14.** Characteristics of Portillo-Gomez and Sosa-Iglesias 2011. | | |  |  |  |  |  |
| --- | --- | --- | --- | --- | --- | --- | --- |
| **Bias** | **Rating** | **Support for judgement** |  |  |  |  |  |
| Recruitment | Probably Low risk | Samples from several hospitals were taken with clinical diagnosis of pulmonary or extra-pulmonary TB (EPTB); unsure if all cases during study period were used |  |  |  |  |  |
| Blinding | Not applicable |  |  |  |  |  |  |
| Confounding | Low risk | Age, sex, nutritional status, PPD, consumption of unpasteurized dairy products, history of contacts and prognosis were recorded for all patients |  |  |  |  |  |
| Exposure assessment | Low risk | Detailed information as above was provided |  |  |  |  |  |
| Incomplete outcome data | Low risk | No indication of incomplete data |  |  |  |  |  |
| Selective reporting | Low risk | No indication of selective reporting |  |  |  |  |  |
| Other bias | Low risk | No other biases are suspected |  |  |  |  |  |
| Conflict of Interest | Probably Low risk | None reported; funding source not stated |  |  |  |  |  |
|  |  |  |  |  |  |  |  |
| Design: | Cross-sectional |  |  |  |  |  |  |
| Participants: | Patients with clinical diagnosis of pulmonary or extra-pulmonary TB | |  |  |  |  |  |
| Exposure: | TB contact, ingestion of unpasteurized cow milk or dairy products; malnutrition | | |  |  |  |  |
| Outcome: | Pulmonary or extra-pulmonary TB | |  |  |  |  |  |
| Notes: | Malnutrition status found in 69% of human cases; significant association between *M.bovis* and EPTB | | | | |  |  |

| **Table S15.** Characteristics of Prasad et al. 2005. | | |  |  |  |  |  |
| --- | --- | --- | --- | --- | --- | --- | --- |
| **Bias** | **Rating** | **Support for judgement** |  |  |  |  |  |
| Recruitment | Probably Low risk | Sampled patients from several clinics and a main medical institute; Did not discuss if all clinically suspected patients were included, or just a subset. Included 56 bovine samples from clinically ill and clinically normal cattle. |  |  |  |  |  |
| Blinding | Not applicable |  |  |  |  |  |  |
| Confounding | Probably Low risk | Analysis addressed differences in laboratory detection; did not address patient differences |  |  |  |  |  |
| Exposure assessment | Low risk | Mycobacterium detection techniques were robust and included sensitivity/ specificity reporting. |  |  |  |  |  |
| Incomplete outcome data | Low risk | No indication of incomplete data set |  |  |  |  |  |
| Selective reporting | Low risk | No indication of selective reporting |  |  |  |  |  |
| Other bias | Low risk | No other potential sources of bias are suspected |  |  |  |  |  |
| Conflict of Interest | Low risk | Funding through Indian government |  |  |  |  |  |
|  |  |  |  |  |  |  |  |
| Design: | Cross-sectional |  |  |  |  |  |  |
| Participants: | Patients clinically suspected of TB seen at various clinics or admitted to the All India Institute of Medical Sciences | | | | | |  |
| Exposure: | Mycobacterium; NOT Livestock exposure | |  |  |  |  |  |
| Outcome: | Extrapulmonary TB in humans and cattle; | |  |  |  |  |  |
| Notes: | 56 cattle; 29 with signs/symptoms of TB and 27 apparently healthy were tested | |  |  |  |  |  |

| **Table S16.** Characteristics of Rahman et al. 2015. | |  |  |  |  |  |  |
| --- | --- | --- | --- | --- | --- | --- | --- |
| **Bias** | **Rating** | **Support for judgement** |  |  |  |  |  |
| Recruitment | Probably Low risk | Patients recruited from one hospital only; enrolled if TB diagnosed by chest radiograph and direct smear positive for AFB |  |  |  |  |  |
| Blinding | Not applicable |  |  |  |  |  |  |
| Confounding | Probably Low risk | Small positive sample size does not alLow for robust statistical adjustment of confounders; they were addressed in univariable analysis |  |  |  |  |  |
| Exposure assessment | Low risk | Livestock risk factors are addressed completely for all human patients; farm level risk factors are addressed for cattle exposure |  |  |  |  |  |
| Incomplete outcome data | Low risk | No indication of incomplete data sets. |  |  |  |  |  |
| Selective reporting | Low risk | All analyses discussed were reported |  |  |  |  |  |
| Other bias | Low risk | No additional biases are suspected. |  |  |  |  |  |
| Conflict of Interest | Low risk | All funding appears to be from government and academia |  |  |  |  |  |
|  |  |  |  |  |  |  |  |
| Design: | Cross-sectional |  |  |  |  |  |  |
| Participants: | TB+ patients admitted at the TB Hospital, Sylhet 3000, Bangladesh; 300 cow milk samples | | | |  |  |  |
| Exposure: | Addressed known risk factors and demographics in people and at farm level for cattle | | | |  |  |  |
| Outcome: | TB diagnosis- *M. bovis* positive | |  |  |  |  |  |
| Notes: | PCR detected *M. bovis* in all samples positive by culture; laboratory techniques were robust | | | |  |  |  |

| **Table S17.** Characteristics of Viegas et al. 2015. | |  |  |  |  |  |  |
| --- | --- | --- | --- | --- | --- | --- | --- |
| **Bias** | **Rating** | **Support for judgement** |  |  |  |  |  |
| Recruitment | Probably Low risk | Author stated 677 patients presented for suspected TBLN during study time; only included 110 presumably because they're they only ones that had FNAs |  |  |  |  |  |
| Blinding | Not applicable |  |  |  |  |  |  |
| Confounding | Probably Low risk | Authors addressed sex, age, HIV status, TB contacts |  |  |  |  |  |
| Exposure assessment | Probably Low risk | Individual raw dairy product use was not characterized; given no incidence of *M. bovis* likely unimportant |  |  |  |  |  |
| Incomplete outcome data | Low risk | No indication of incomplete outcome data |  |  |  |  |  |
| Selective reporting | Low risk | No indication of selective reporting |  |  |  |  |  |
| Other bias | Low risk | No other biases were indicated |  |  |  |  |  |
| Conflict of Interest | Low risk | Funded by government agency |  |  |  |  |  |
|  |  |  |  |  |  |  |  |
| Design: | Cross-sectional |  |  |  |  |  |  |
| Participants: | Suspected TBLN patients in Maputo, Mozambique Jul2013-Jul 2014 | |  |  |  |  |  |
| Exposure: | Mycobacteria |  |  |  |  |  |  |
| Outcome: | TBLN |  |  |  |  |  |  |
| Notes: | No *M.bovis* found; presumed to be because population was periruban/ urban, so Low risk | | | |  |  |  |

**Table S18.** Data extraction for included studies.
